# Final results of the Choroid Plexus Tumor study CPT-SIOP-2000

**DOI:** 10.1007/s11060-021-03942-0

**Published:** 2022-01-08

**Authors:** Johannes E. Wolff, Stefaan W. Van Gool, Tezer Kutluk, Blanca Diez, Rejin Kebudi, Beate Timmermann, Miklos Garami, Jaroslav Sterba, Gregory N. Fuller, Brigitte Bison, Uwe R. Kordes

**Affiliations:** 1 University of Regensburg, Regensburg, Germany; 2 Present Address: Oncology Development, AbbVie, Chicago, IL USA; 3 IOZK Immune Oncological Centre Cologne, Cologne, Germany; 4 Department of Pediatric Oncology, Hacettepe University Medical School and Cancer Institute, Ankara, Turkey; 5 Neuro Oncology Program, Institute of Neurological Research, FLENI, Buenos Aires, Argentina; 6 Pediatric Hematology-Oncology, Oncology Institute, Istanbul University, Istanbul, Turkey; 7 Department of Particle Therapy, University Hospital Essen, West German Proton Therapy Centre Essen (WPE), West German Cancer Center (WTZ), German Cancer Consortium (DKTK), Essen, Germany; 8 2nd Department of Pediatrics, Semmelweis University, Budapest, Hungary; 9 Department of Pediatric Oncology, University Hospital Brno and Faculty of Medicine, Masaryk University, Brno, Czech Republic; 10 International Clinical Research Center, St. Anne’s University Hospital, Brno, Czech Republic; 11 Departments of Neuropathology and Neuroradiology, MD Anderson Cancer Center, Houston, USA; 12 Department of Neuroradiology, University Hospital of Augsburg, Augsburg, Germany; 13 Department of Pediatric Hematology and Oncology, University Medical Center Hamburg, Hamburg, Germany

**Keywords:** Choroid plexus tumors, Chemotherapy, Irradiation, Li–Fraumeni syndrome

## Abstract

**Introduction:**

Standards for chemotherapy against choroid plexus tumors (CPT) have not yet been established.

**Methods:**

CPT-SIOP-2000 (NCT00500890) was an international registry for all CPT nesting a chemotherapy randomization for high-risk CPT with Carboplatin/Etoposide/Vincristine (CarbEV) versus Cyclophosphamide/Etoposide/Vincristine (CycEV). Patients older than three years were recommended to receive irradiation: focal fields for non-metastatic CPC, incompletely resected atypical choroid plexus papilloma (APP) or metastatic choroid plexus papilloma (CPP); craniospinal fields for metastatic CPC/APP and non-responsive CPC. High risk was defined as choroid plexus carcinoma (CPC), incompletely resected APP, and all metastatic CPT. From 2000 until 2010, 158 CPT patients from 23 countries were enrolled.

**Results:**

For randomized CPC, the 5/10 year progression free survival (PFS) of patients on CarbEV (n = 20) were 62%/47%, respectively, compared to 27%/18%, on CycEV (n = 15), (intention-to-treat, HR 2.6, p = 0.032). Within the registry, histological grading was the most influential prognostic factor: for CPP (n = 55) the 5/10 year overall survival (OS) and the event free survival (EFS) probabilities were 100%/97% and 92%/92%, respectively; for APP (n = 49) 96%/96% and 76%/76%, respectively; and for CPC (n = 54) 65%/51% and 41%/39%, respectively. Without irradiation, 12 out of 33 patients with CPC younger than three years were alive for a median of 8.52 years. Extent of surgery and metastases were not independent prognosticators.

**Conclusions:**

Chemotherapy for Choroid Plexus Carcinoma is feasible and effective. CarbEV is superior to CycEV. A subset of CPC can be cured without irradiation.

**Supplementary Information:**

The online version contains supplementary material available at 10.1007/s11060-021-03942-0.

## Introduction

Choroid plexus tumors (CPT) are rare brain tumors of the choroid plexus epithelium. The age-standardized incidence rate is 1.0 per million, with an incidence peak in the first year of life at 6.1 per million [1]. The WHO classification differentiates between low-grade choroid plexus papilloma (CPPCNS WHO grade 1), intermediate-grade atypical choroid plexus papilloma, characterized by increased mitotic activity (APPCNS WHO grade 2), and high-grade choroid plexus carcinoma, which displays frank signs of malignancy (CPCCNS WHO grade 3) [2]. DNA methylation profiling further segregates three distinct subclass: supratentorial pediatric low-risk CPT (CPP/APP) = “pediatric A”, infratentorial adult low-risk CPT (CPP/APP) = “adult”, and supratentorial pediatric high-risk CPT (all CPC, very few APP/CPP) = “pediatric B” [3–6]. CPC is the typical CPT seen in Li-Fraumeni Syndrome [7].

Treatment recommendations for CPT include multidisciplinary approaches, with maximal surgical resection for all CPT [8–14], followed by chemotherapy [11, 15–21] and radiotherapy [22–24] for high-risk CPT. The prognosis of CPC remains dismal when tumor resection is the only treatment modality, and the role, sequence, and intensity of primary chemotherapy remain debatable [13, 19, 21].

We here report the registry results, and the final results of the first global trial for CPT, which was designed in the late 1990s by an international multidisciplinary pediatric neurooncology collaboration following a metaanalysis [22, 25].

The aims were (a) to initiate a registry for the prospective collection of CPT data, (b) to design a multidisciplinary treatment algorithm supporting clinical care by using information from single cases and small series [22, 23, 26], and (c) to perform a randomized interventional study comparing six cycles of carboplatin/etoposide/vincristine (CarbEV) versus cyclophosphamide/ etoposide/vincristine (CycEV).

## Methods and materials

CPT-SIOP-2000 (NCT00500890) was approved by the SIOP scientific committee, the leading institution ethics committee (Regensburg, Germany), local institutional ethics committees, and the German Cancer Society in 2000. Written informed consent was obtained from patients, parents, or appropriate legal guardians in accordance with national laws.

### Registry

**Fig. 1:**
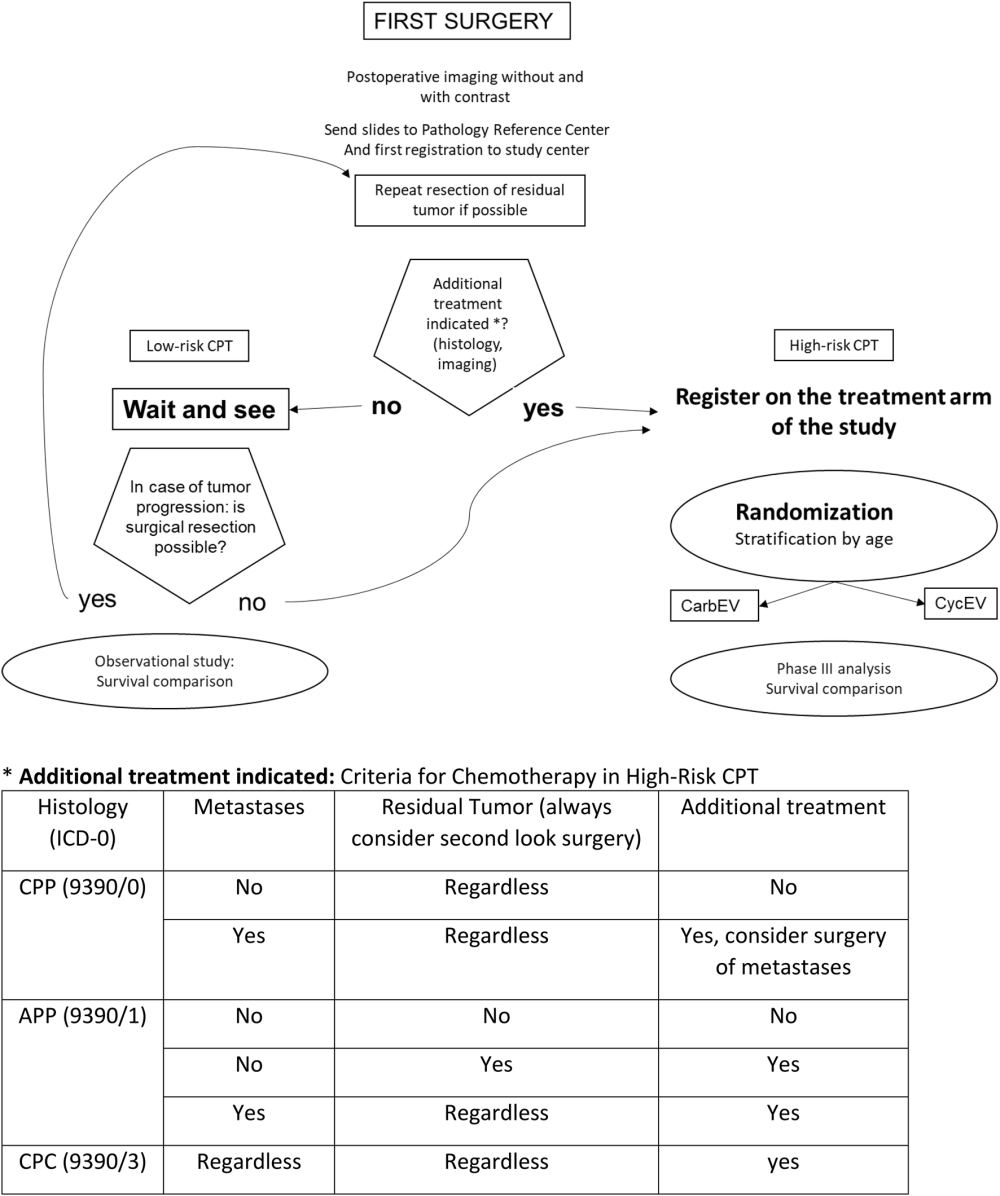
CPT-SIOP-2000 algorithm for surveillance and intervention allocation. The original flow chart shows the overall design of the observational registry for low-risk CPT and the interventional chemotherapy study for high-risk CPT. High-risk CPT criteria are listed. These defined the indications for chemotherapy with randomized CarbEV and CycEV (Supplemental Fig. 2) and radiotherapy, with separate indications for volumes and doses (Supplemental Table 3). The protocol design did not include cross-over between CarbEV and CycEV arms for non-responders

Patients with histologically-confirmed newly-diagnosed CPT were eligible for registration, which included all ages, performance status, tumor grade and metastatic status (eligibility criteria listed in Table 1a). Central histology and radiology reviews, as well as Li–Fraumeni syndrome (LFS) testing, were recommended, but not mandatory. Figure 1 depicts the algorithm of registry surveillance for low-risk CPT (non-metastatic CPP and completely resected non-metastatic APP). Data from patients receiving non-protocol therapy were also collected.

**Table 1:** CPT-SIOP-2000 inclusion and exclusion criteria, outcome, performance status

(a) Eligibility Criteria for Registry	
Inclusion	(1) Local diagnosis of CPT
	a. Choroid plexus papilloma (ICD-O 9390/0)
	b. Atypical choroid plexus papilloma (ICD-O 9390/1)
	c. Choroid plexus carcinoma (ICD-O 9390/3)
	(2) Slides sent for pathology reference review
Exclusion	(1) Patient or legal guardian does not consent to enrollment with electronic data processing or sending of tumor slides to the pathology reference center

(b) Eligibility Criteria for Randomized Study Intervention Chemotherapy	
Inclusion	(1) The first registration on the study was completed
	(2) The pathology reference center has confirmed the receipt of histological slides
	(3) Postoperative MRI imaging has been performed and the results are available
	(4) Any of the High-Risk CPT criteria are met (Fig. 1)
	(5) The chemotherapy start criteria^a^ are met
	(6) The agreement of the patient or legal guardian has been documented according to local guidelines
Exclusion	(1) Previous irradiation or chemotherapy
	(2) Patient or legal guardian does not agree with treatment or randomization
	(3) Clinical start criteria for the planned treatment as outlined in treatment modification guidelines are not met
	(4) The protocol did not pass the local center required approvals, such as Ethics Committee or scientific review
	(5) Previous antiangiogenic therapy
	(6) Previous immunotherapy

(c) Objectives and outcome definitions	
*Survival times*	
Primary Objective: Overall Survival (OS)	Time from histological diagnosis until death, or the date last seen (censored)
Secondary Objective: Progression Free Survival (PFS)	Time from histological diagnosis to disease progression or death, or the date last seen (censored)
Event Free Survival (EFS)	Time from histological diagnosis until tumor progression, second malignancy, death, or the date last seen (censored)
*Response evaluation*	
Complete response (CR)	No evidence of tumor
Partial response (PR)	Remaining evidence of tumor, with tumor size in cross-sectional area ≤ 50% of pretreatment value in all known tumor locations;
Stable disease (SD)	Tumor size > 50% and ≤ 125%
Progressive disease (PD)	Tumor size > 125% of pretreatment value in any individual tumor location or new lesion

(d) Performance status	
Level 1	normal activity, no disabilities
Level 2	minor disability, not requiring additional assistance
Level 3	age-related activity greatly reduced
Level 4	bed-ridden, requiring nursing care
Level 5	intensive medical care, moribund

WHO definitions [2]. ∙ Choroid plexus papilloma: Delicate fibrovascular connective tissue fronds are covered by a single layer of uniform cuboidal to columnar epithelial cells with round or oval, basally situated monomorphic nuclei. Mitotic activity is extremely low. Brain invasion, high cellularity, necrosis, nuclear pleomorphism and focal blurring of the papillary pattern are unusual, but may occur. CPP closely resembles non-neoplastic choroid plexus, but cells tend to be more crowded, elongated or stratified instead of the normal cobblestone-like surface. ∙ Atypical choroid plexus papilloma: A choroid plexus papilloma with increased mitotic activity (≥ 1 mitosis/mm^2^; equating to ≥ 2 mitoses per 10 randomly selected high power field of each 0.23 mm^2^). Up to two of the following four features may be present, but are not required: increased cellularity, nuclear pleomorphism, blurring of the papillary pattern, areas of necrosis

Choroid plexus carcinoma: Malignant epithelial neoplasm of the choroid plexus that shows at least four of the following five features: frequent mitoses, increased cellular density, nuclear pleomorphism, blurring of the papillary pattern with poorly structured sheets of tumour cells, necrotic areas

^a^*Chemotherapy start criteria* White blood cell count: > 2000/μl; platelet count: > 85,000/μl; serum creatinine: in normal range; pregnancy test: negative (women of childbearing potential); audiology: hearing loss less than 30 dB at 3000 Hz

### Interventional study

Patients with either CPC, metastatic disease, or incompletely resected APP were eligible for randomized chemotherapy intervention (Fig. 1; eligibility criteria listed in Table 1b). Open label randomization was provided by the study center. Six cycles of chemotherapy were repeated every 28 days and consisted of etoposide 100 mg/msq on days 1–5, with vincristine 1.5 mg/msq on day 5. The third drug was randomized to either carboplatin 350 mg/msq on days 2 and 3 (Supplemental Fig. 1a: CarbEV) or cyclophosphamide 1 g/msq on days 2 and 3 (Supplemental Fig. 1b: CycEV). Radiotherapy was proposed after two cycles of chemotherapy and restricted to patients that were at least 3 years of age: local fields with 54 Gy administered in 30 fractions (1.8 Gy/fraction) were prescribed for non-metastatic CPC, APP with residual tumor and metastatic CPP. Craniospinal fields of 35.2 Gy in 22 fractions (1.6 Gy/fraction) with a local boost of up to a total of 54 Gy for primary tumor and 49.6 Gy for metastases (both with 1.8 Gy/fraction), were prescribed for patients with metastatic CPC and APP (Supplemental Table 1a, b).

Feasibility of the study was tested in a pilot phase completed in 2005. The primary objective of the trial was Overall Survival (OS) time (Table 1c). Performance status at diagnosis was graded on a 1–5 level scale (Table 1d). Toxicity was documented in a study-specific grading system (Supplemental Table 2). Statistical analyses were performed in SPSS version 18 (IBM), and GraphPad Prism version 7.00 (GraphPad Software, La Jolla, California, USA, www.graphpad.com). Survival curves were estimated by the Kaplan–Meier method and compared between histologies. Overall survival (OS) was calculated from time of histological diagnosis until death. Event free survival (EFS) was calculated from time of histological diagnosis until tumor progression, second malignancy, death, or date last seen (censored). Progression free survival (PFS) was calculated from time of histological diagnosis to disease progression, death, or date last seen (censored).

## Results

### Registry (Consort Diagram 1, Fig. 2a)

**Fig. 2:**
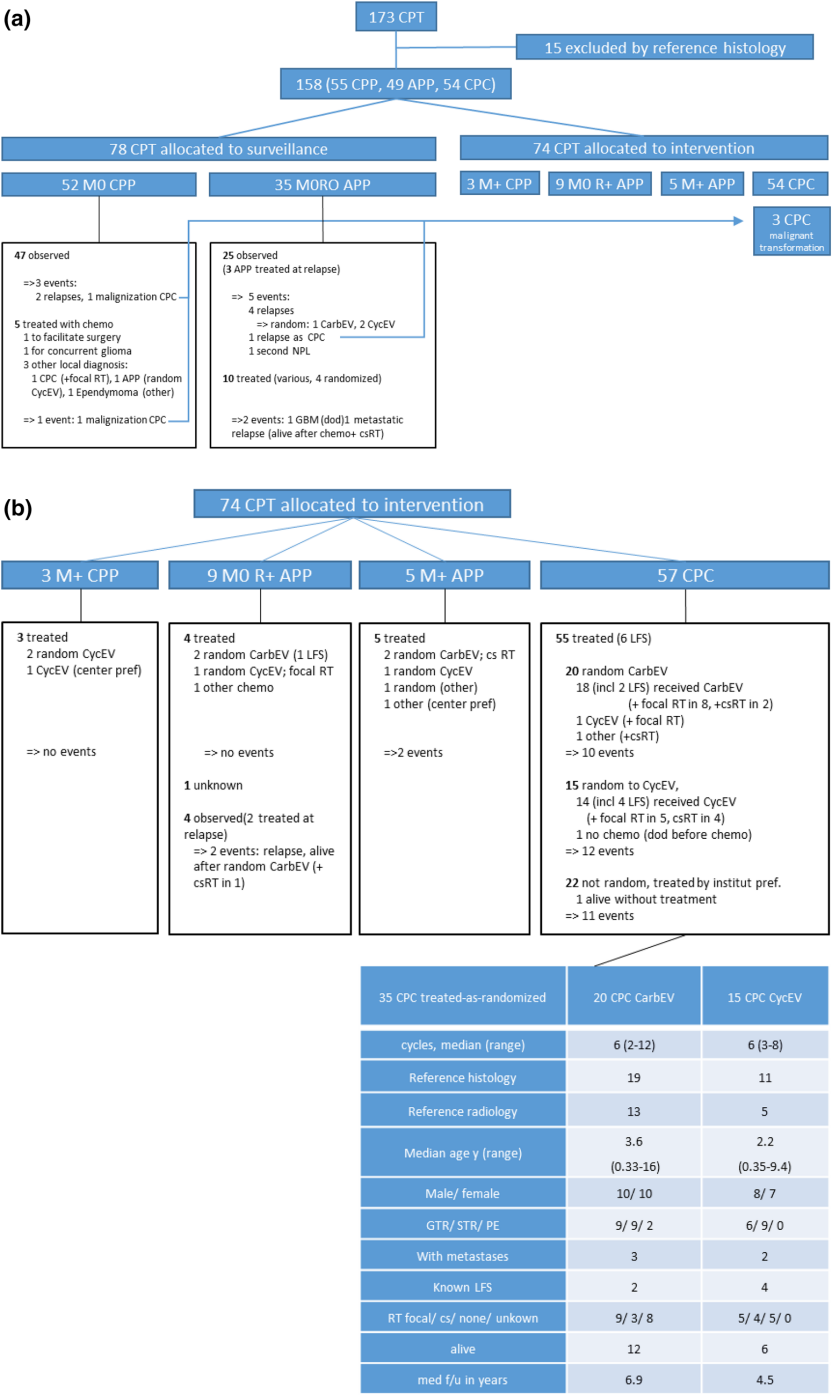
a Consort Diagram 1: Enrollment, exclusion, and allocation to surveillance according to protocol risk stratification (low-risk *versus* high-risk CPT) are shown. Out of 173 screened patients 158 were eligible, with reference histology performed in 138 and reference radiology in 43. 87 patients were allocated to registry surveillance. In three reference-reviewed cases, malignant transformation to CPC occurred, indicated by the thin blue arrows resulting in transfer to the intervention allocation; none of these were randomized. 47 of 52 CPP staged M0 underwent surveillance, and 3 events occurred in this group: 1 malignant transformation, 1 relapsed patient treated by surgery, and 1 relapsed treated by surgery and off-study secondary chemotherapy. 5 of 52 CPP staged M0 received primary off-study chemotherapy: 1 to successfully facilitate surgery, 3 at the investigator’s discretion because of malignant local pathology, and 1 because of a concurrent malignant glioma. 25 of the 35 APP staged M0R0 underwent surveillance; 5 events occurred in this group: 3 local non-metastatic relapses that received on-study chemotherapy and additional focal RT in 1; 1 relapse and malignant transformation treated with surgery, off-study chemotherapy and csRT; and 1 subsequent neoplasm (ameloblastoma). 10 of 35 APP staged M0R0 received chemotherapy at the investigator’s discretion because of malignant local histology; 2 events occurred in this group: 1 secondary GBM, 1 metastatic relapse. *APP* Atypical Choroid Plexus Papilloma, *CarbEV* carboplatin/etoposide/vincristine, *CPC* Choroid Plexus Carcinoma, *CPT* Choroid Plexus Tumor, *CPP* Choroid Plexus Papilloma, *csRT* craniospinal radiotherapy, *CycEV* cyclophosphamide/etoposide/vincristine, *dod* dead of disease, *LFS* Li-Fraumeni Syndrome, *M*+ presence of metastasis, *M0* no metastasis, *pref* preference, *R*+ residual tumor (partial resection or biopsy), *random* randomized, *R0* no residual tumor after tumor surgery, *RT* radiotherapy. b Consort Diagram 2: Allocation to Intervention. Diagram shows patient allocation to treatment intervention according to protocol risk stratification. 3 CPP staged M+ received primary chemotherapy, 2 randomized for CycEV, 1 received off-study CycEV; no events occurred in this group. 9 APP were staged M0R+, 3 received study-chemotherapy, with additional focal RT in one, 1 received off-study chemotherapy; there were no events in this group. 4 APP staged M0R + were observed at the investigator’s discretion; there was one local relapse treated with chemotherapy alone, and one metastatic relapse treated with chemo and csRT. 5 APP staged M + were all treated with chemotherapy; three received randomized chemo, and two received off-study chemotherapy. Two events occurred in this group (PD). 57 CPC, including three secondary CPC after malignant transformation, were dispositioned to intervention. 1 patient died before chemotherapy; 1 patient is alive without non-surgical treatment. The intention-to-treat analysis comprises 35 CPC as-intended (CarbEV 20, CycEV 15). Relevant demographic variables were distributed homogeneously, as shown in the bottom text-box. A total of 9 APP (5 at diagnosis and 3 APP at relapse in surveillance) were also treated-as-randomized (CarbEV 6, CycEV 3)

173 patients were screened from 85 institutions across 23 countries from 05-Jan-2000 until 22-Jan-2010. The database was locked for this analysis in 2020. Central review excluded 15 tumors from the analysis as non-CPT (4 ATRT, 2 low-grade glioma, 1 ependymoma, 2 medulloepithelioma, 2 pineal non-CPT, 1 cribriform neuroepithelial tumor, 3 undeterminable). CPT were upgraded in 12 and downgraded in 17 cases. In 3 cases a local non-CPT diagnosis was revised to CPT. After this review, 158 patients (77 females, 81 males) were included for further analyses, median follow-up for these were 7.4 (0.2–17) years; pathology central review was available in 138 patients (further details: Fig. 2a, b).

The median age at diagnosis for all patients was 1.7 years (0.01–45.6); that for patients with CPP (n = 55) was 2.7 years, for APP (n = 49) 0.7 years, and for CPC (n = 54) 2.1 years. Demographical and clinico-pathological variables are summarized in Table 2. Performance status on the 5-level scale was documented in 46 patients: 20% were in level 1, 56% in level 2, 22% in level 3, and 2% in level 4 or 5. Values did not correlate with histology or outcome.

**Table 2:** Patient demographics

Primary histology	CPP	APP	CPC	Total
Number of patients	55	49	54	158
Female/male	26/29	24/25	27/27	77/81
Median age at diagnosis in years (range)	2.6 (0.2–46)	0.7 (0.01–13)	2.1 (0.3–18)	1.7 (0.01–46)
Pathogenic germline *TP53* variation (LFS)	–	1	6	7
Screening for LFS performed	–	2	7	9
Sotos syndrome		1		
Median tumor volume in ml (range), number of patients	38 (5–302) 7	71 (11–231) 16	50 (12–415) 22	53 (5–415) 45
Primary location: lateral ventricle, n (%)	35 (64%)	41(84%)	51 (94%)	127 (80%)
IIIrd ventricle, n (%)	4 (7%)	4 (8%)	1 (2%)	9 (6%)
IVth ventricle, n (%)	14 (26%)	3 (6%)	2 (4%)	19 (12%)
Other (IIIrd + IVth; CPA)	2 (3%)	1 (2%)		3 (2%)
Primary metastases, n (%)	3 (6%)	5 (10%)	11 (20%)	19(12%)
Subsequent neoplasms *multiple neoplasm in same patient ^†^LFS confirmed by testing [n] number of treatment exposures prior to first subsequent neoplasm	1 brainstem glioma [0]	1 ameloblastoma [0] 1 GBM * & 1 STS * [2] 1 RMS [2] 1 hemangioma [0]	1 AML/MDS [3] 1 AML/MDS [2] 1 AML* & 1 nephroblastoma*^†^ [2] 1 epithelioma [4] 1 skull base tumor (suspected meningioma/neurinoma) [2]	12 in 10 patients

For medium tumor volume calculations, the ellipsoid volume formula was used: 4/3 π [A/2 × B/2 × C/2]), where A, B and C are the maximum dimensions in the standard planes: axial (cranio-caudal, A), coronal (transverse, B) and sagittal (anteroposterior), results corresponded well with the abridged ellipsoid formula (1/2 (A × B × C)) as used by the SIOPE Imaging protocol for patients in European SIOP Brain Tumour Studies. In 27 of 45 tumor volumes calculations reference radiology was available

*APP* Atypical Choroid Plexus Papilloma, *CPA* cerebellopontine angle, *CPC* Choroid Plexus Carcinoma, *CPP* Choroid Plexus Papilloma, *GBM* glioblastoma multiforme, *LFS* Li-Fraumeni Syndrome, *STS* soft tissue sarcoma, *multiple neoplasms in the same patient, ^†^LFS confirmed by molecular analysis, [n] number of treatment exposures before first subsequent neoplasm, chemotherapy and radiotherapy are counted separately

LFS testing was performed for only 9 patients, which was prompted by positive family history in three, and detected pathogenic *TP53* mutations in 1 APP (c.743G>A; p.R248Q) and 6 CPC (c.818G>A, p.R273H; codon 170 4 bp del leading to stop in codon 173; c.847C>T, p.R283C; c.356C>G, pA119G; c.742C>T, p.R248W; mutation not communicated in one). LFS was suspected, but not tested, in one patient with CPC who developed subsequent glioblastoma and malignant hemithorax tumor, and in another patient with APP who had a previous periorbital rhabdomyosarcoma.

Sotos syndrome was diagnosed in one patient with APP. Other co-morbidities were univentricular heart in 1 CPC patient, ureteral duplication in 1 CPP patient, ectopic kidney in 1 APP patient, and demyelinating disease in 1 APP patient.

Available tumor volumes did not differ amongst the CPT subgroups in this cohort (median for all = 53 ml). 80% of all CPT were located in the lateral ventricles (94% of CPC, 84% of APP, 64% of CPP); location in the fourth ventricle was more common in older patients (15/19 CPT > 3 years versus 4/19 CPT ≤ 3 years), and less frequent for high-grade tumors (4% of CPC, 6% of APP, 26% of CPC). Only 1 CPC arose in the third ventricle (Table 2).

At initial staging, 134 patients had localized disease, and 19 had metastatic disease (3 CPP, 5 APP, 11 CPC). Of the 19 patients with metastatic disease, 2 were identified on the basis of positive CSF cytology only, 1 patient had intracranial metastases, 8 patients had spinal metastases, and 6 patients had generalized leptomeningeal disease. For 2 patients with metastatic disease the location was not documented. Staging data were not available in 5 patients. Non-metastatic tumors were more commonly completely resected.

**Fig. 3:**
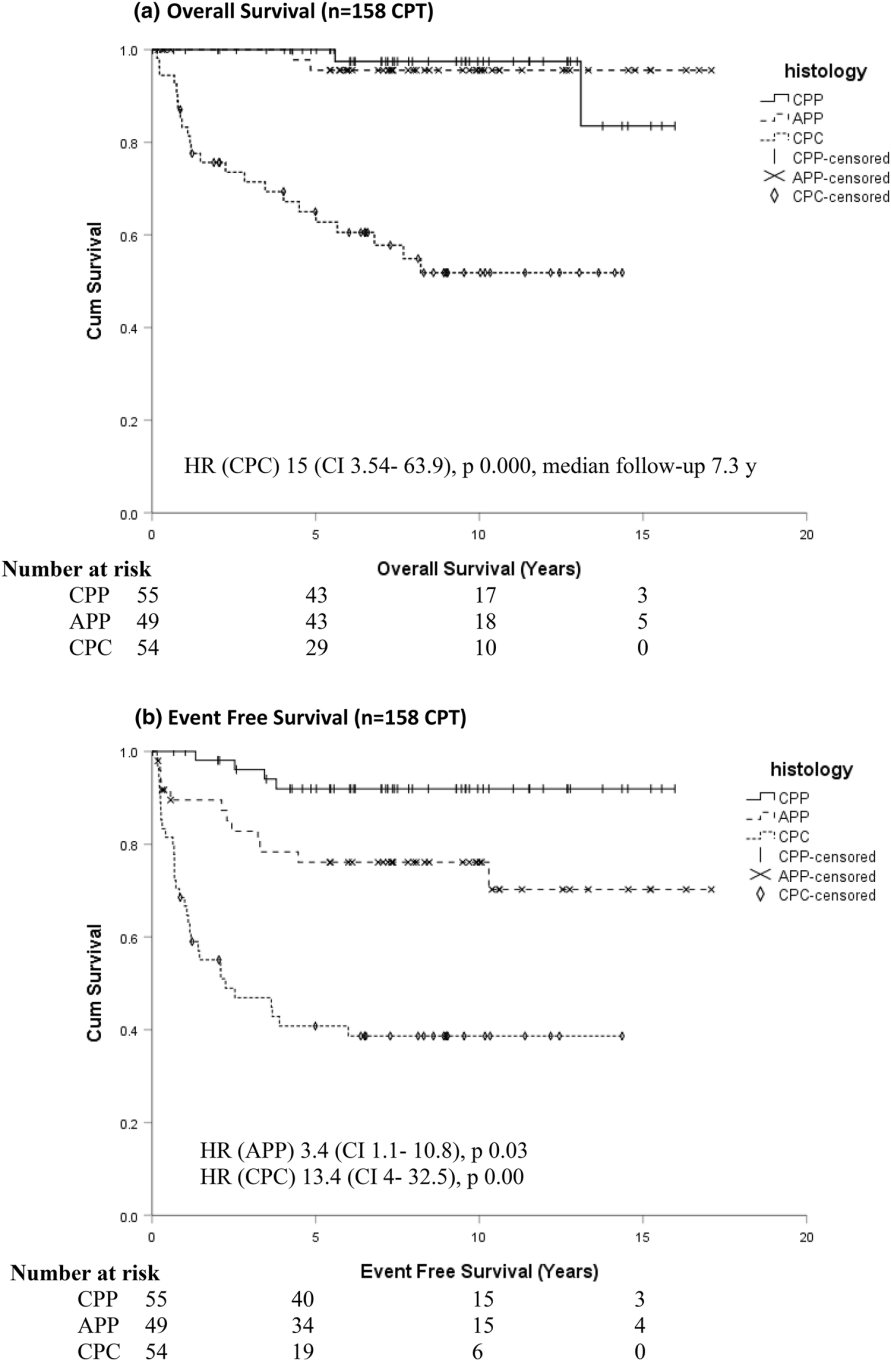

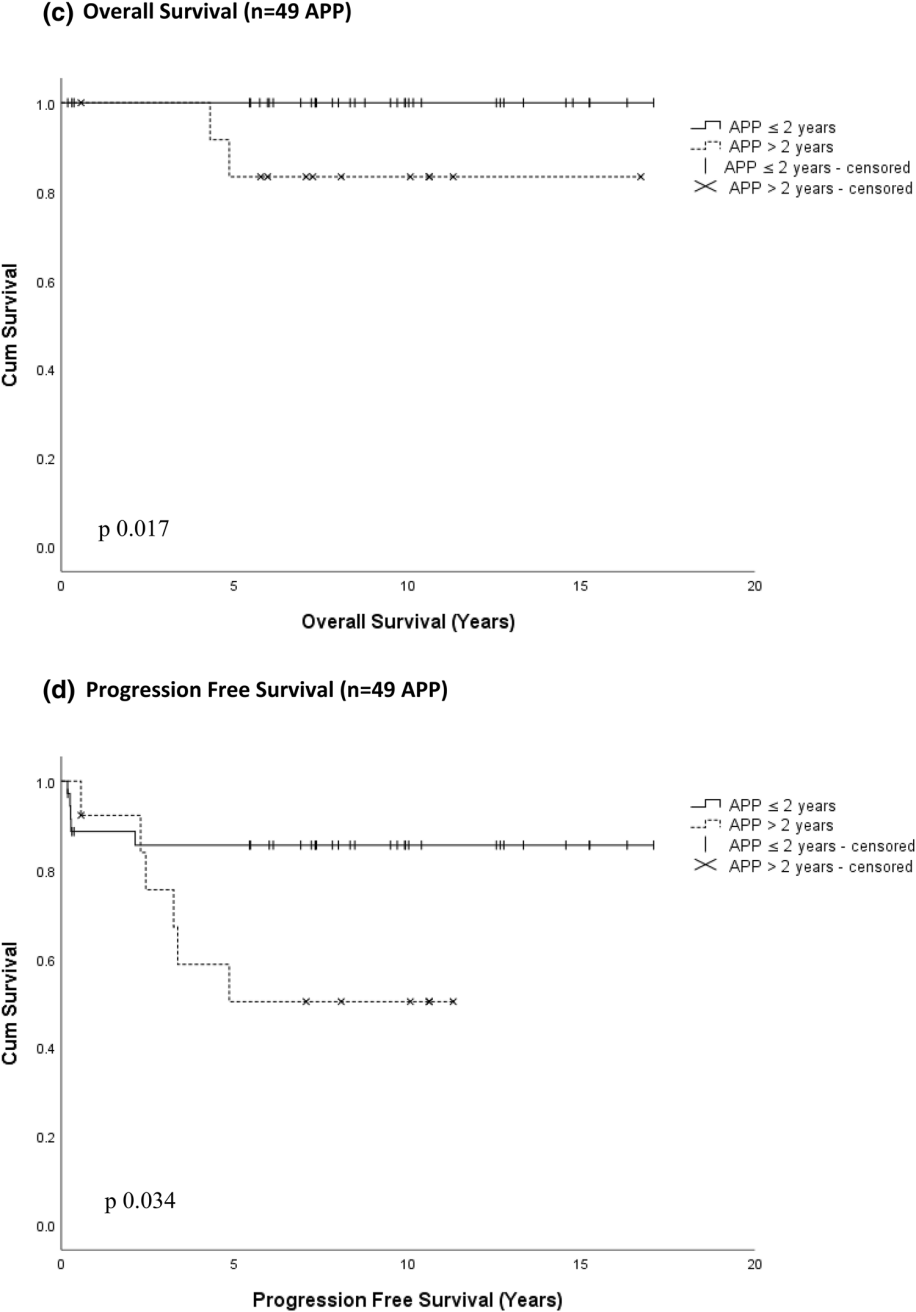
Overall survival (**a**) and event free survival (**b**) of all 158 patients registered to CPT-SIOP-2000 by histology. Pathology central review was missing in 20 patients: 5 CPP, 3 APP, 12 CPC. 4 of these 12 non-referenced CPC were randomized and treated with CycEV and one was treated with CarbEV; 1 of 3 non-referenced APP was randomized and treated in CarbEV. Three patients with malignant transformation that was detected at surgery for relapse are included here with their histology grading at primary diagnosis. This has particular impact on the CPP curves. Two patients with an original diagnosis of CPP had an increase in tumor grade before treatment was initiated, and died later. If the curves were generated taking only the histology at treatment start into account, then there would be no deaths in the CPP curve. One patient with APP also had malignant transformation. Age effect for Overall Survival (**c**) and Progression Free Survival (**d**) in 49 patients with APP, pathology central review missing in 3 APP. *APP* Atypical Choroid Plexus Papilloma, *CPC* Choroid Plexus Carcinoma; *CPP* Choroid Plexus Papilloma, *HR* Hazard Ratio, *CI* Confidence Interval

Histological grade was the single most prominent prognostic variable (Fig. 3a,b). The 1, 5, and 10 year OS were as follows: CPP (n = 55): 100%, 100%, 97%; APP (n = 49): 100%, 96%, 96%; CPC (n = 54) 83%, 65%, 51%. The 1, 5, 10 year EFS were: CPP: 100%, 92%, 92%; APP: 90%, 76%, 76%; and CPC: 68%, 41%, 39%. Two patients with CPP experienced malignant progression to CPC and died of progressive disease 5.6 and 13.1 years after primary diagnosis, respectively. One instance of malignant progression occurred in an APP patient. APP patients younger than 2 years of age at diagnosis had significantly higher higher PFS and OS compared to older APP patients (Fig. 3c, d).

For 86 CPT (30 CPP, 25 APP, 31 CPC) a nuclear p53 labeling index was determined and subsequently correlated with grade: 2/30 CPP were positive (index 10%), 6/25 APP were positive (index ≤ 30%), and 19/31 CPC were positive (index 10–90%). All CPT with a p53 labeling index over 35% were CPC. Nuclear p53 labeling was assessed in 4 of 7 patients with LFS: the index was 0% in 2 patients, 10% in one patient, and 50% in one patient. There was no prognostic relevance of p53 labeling within any of the histological groups. Methylation profiling [3, 5, 6] was available for 36 patients, with classification results as follows: pediatric A in 9 (4 APP, 5 CPP); pediatric B in 25 (3 CPP, 8 APP, 14 CPC); and adult in 2 CPP. One of the three low-grade reference-reviewed CPT with subsequent malignant transformation was a CPP that classified into the high-risk pediatric subgroup B by methylation profiling.

### Surgery

Complete resection was documented in 98 patients, partial resection in 56, and biopsy in 4 patients. Extent of surgery did not correlate with demographic variables or primary tumor location, but complete resection was achieved less frequently in CPC compared to APP and CPP (42% versus 69% and 77%, respectively: p = 0.0032), and the average size of completely resected tumors was significantly smaller than those which were only partially resected: 34 cm^3^ (range 0.5–184) versus 76 cm^3^ (range 12–415) (Mann Whitney test: p = 0.003). The prognostic impact of complete resection on survival of all CPT appeared significant for EFS and borderline for OS: the five-year EFS and OS rates were 82% (CI95% 72–88) and 89% (CI95% 80–94) versus 64% (CI95% 50–75) and 81% (CI95% 68–89) after complete resection *versus* less than total resection. However, these differences were confounded by the histological grade. When analyzing within each histological group, there was no significant benefit from surgery for PFS, EFS or OS (for CPC: Supplemental Fig. 2).

### Radiotherapy

Following at least two cycles of chemotherapy, 30 patients with CPC and 8 with APP received irradiation. One child with CPP was irradiated because of a local diagnosis of CPC. Eleven patients with CPC received craniospinal irradiation plus local boost (median age at irradiation: 5.9 years, range 3–21.2). Nineteen received local radiotherapy (median age 5.6 years, range 1.5–18.6). The 2-year EFS without and with radiotherapy was 47% (CI95% 27–65) versus 76.5% (CI95% 56.9–88, p = 0.23), respectively. The 2-year OS without and with radiotherapy was 55% (CI95% 34–72) versus 96.7% (CI95% 78.7–99.5), respectively. This difference did not reach statistical significance (Log-rank test: p = 0.052). Among APP patients, for irradiated *versus* not irradiated, the 5 year OS/PFS was 75%/63% *versus* 100%/92%, respectively.

### Chemotherapy in high-risk CPT (Consort Diagram 2, Fig. 2b)

Chemotherapy was provided to 87 CPT comprising 8 CPP, 24 APP, and 55 CPC (including the three CPC arising via malignant transformation): 35 CarbEV, 41 CycEV, 11 other (site decision). 6 of 18 CPP/APP with incomplete resection had an OR (CR + PR) after two cycles of chemotherapy. 35 patients started CarbEV or CycEV after complete resection; none of these experienced tumor progression during the first two cycles. Twelve of 33 patients with CPC younger than 3 years of age at diagnosis were treated with chemotherapy only (without radiation) and are alive with a median follow-up of 8.52 years (0.86–12.79).

### Carboplatin versus cyclophosphamide randomization

As per intention-to-treat (ITT) 20 CPC were randomized for CarbEV and 15 for CycEV. The study arms had matching clinical values gender (equal), extent of resection (GTR in 50% each), metastases (n = 3 in CarbEV, n = 2 in CycEV), radiotherapy (60% *versus* 64%); median age was higher in CarbEV (3.6 y, 0.22–16) vs CycEV (2.1 y, 0.35–9.4), there were 2 cases with LFS in the CarbEV and 4 in the CycEV arm%). After two cycles of chemotherapy the response of 17 CPC with incomplete resection was 1 CR, 4 PR, 7 SD, 3 PD, and 2 NA; the ORR (CR + PR) here was 55% in CarbEV (n = 9) versus 0% in CycEV (n = 8) (p < 0.05, Fisher Exact Test, treated as randomized group), and none of the 15 completely resected tumors had recurred.

**Fig. 4:**
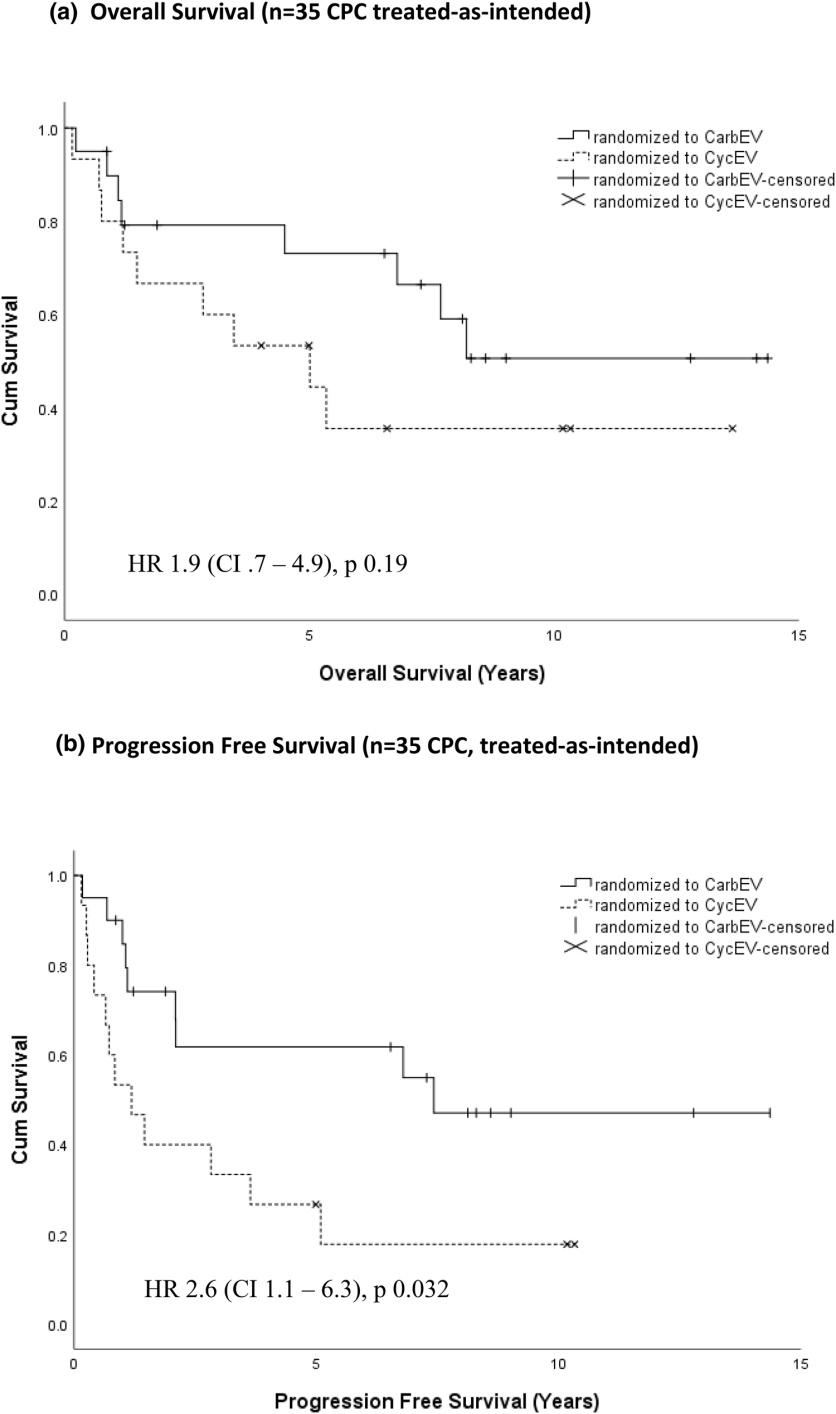
Overall survival (**a**) and progression free survival (**b**) by chemotherapy arm for 35 CPC patients as per intention-to-treat, CarbEV-arm (n = 20) or CycEV-arm (n = 15). Pathology central review missing in 5 CPC. Results for treated-as-randomized (CarbEV n = 18; CycEV n = 14 are very similar)

The 5/10 year OS and PFS as per ITT for CarbEV was 73%/51% and 62%/47%, respectively, with 12 alive, compared to 53%/36% and 27%/18%, respectively, for CycEV (HR 2.6, p = 0.032 for PFS), with six alive (Fig. 4).

### Safety

Chemotherapy with CarbEV/CycEV was tolerable—within expected range and without treatment-related deaths. Grade 4 toxicity was limited to leukopenia and thrombopenia. All adverse event data are summarized in Supplemental Table 2. Second malignancies were common: 12 subsequent neoplasms were documented in 10 patients (Table 2): one ameloblastoma 10 years after APP; three myeloid malignancies (2.5, 6 and 3.9 years after CPC), one with an additional nephroblastoma; a glioblastoma 4 years after APP, followed by a soft tissue sarcoma in the same patient (died from glioblastoma); a brainstem astrocytoma with a CPP; one rhabdomyosarcoma prior to APP; one epithelioma after CPC (died from CPC); one hemangioma after APP; and one skull base tumor (radiological meningioma/neurinoma) 1.2 years after CPC. All patients with myeloid malignancies died, two of these from treatment-related complications after intensive chemotherapy.

## Discussion

We report here the largest prospective and the only randomized trial for choroid plexus tumors published to date. The long median observation time of 7.3 years is one outstanding feature. Histological grade emerged as the most relevant prognostic factor, and the value of CarbEV was established for choroid plexus carcinoma. Limitations are incomplete reference review for histology and radiology, and incomplete molecular work-up.

CPP are low-grade tumors with a very high OS; however, they do not completely follow all characteristics of a benign tumor. While mostly localized, typically in the fourth ventricle, three were metastatic at the time of diagnosis, two of these confirmed as by reference radiology. All three received primary chemotherapy and are alive without event at 8.4, 10 and 14.4 years, with PR, SD, and CR, respectively, after 2 cycles. Two untreated CPP, one with methylation subclass pediatric B, progressed to CPC, which is a recognized, albeit rare, event [27]. Both patients received salvage treatment but died from PD 3.1 and 9.3 years after primary diagnosis.

In contrast to CPP, half of the patients with CPC died despite intensive treatment (Fig. 3a). The study data solidify known demographics (Table 1): patients were young (median age 2.1 years), without gender predominance, CPC were mostly located in the lateral ventricles, and 3/57 of the available family histories were positive for LFS, which is relevant for counselling and treatment choices [28]. The prevalence of de novo LFS is known to be high in CPC [7], but due to limitations of the study this could not be fully addressed. Among the detected *TP53*-germline mutations, one novel complex deletion was identified; the others confirmed previous listings in COSMIC and IARC TP53. The occurrence of an APP in Sotos syndrome described here is a novel finding, expanding the spectrum of this *NSD1-*related over-growth and tumor-predisposition [29].

Staining for p53 in this study correlated with histology: all CPT with a labeling index > 30% were CPC. However, this finding was without independent prognostic relevance, apparently contradicting previous reports [30]. The discrepancy might be explained by the integration of histological grade in this analysis (without the covariate, p53 was a negative prognostic variable in this study as reported in others), or by the laboratory technique. Two CPC with underlying LFS had absent staining for p53. Taken together, the data show the limitations of using p53 immunohistochemistry for informing treatment stratification.

This study expands findings from our previous analysis of APP patients [20]. PFS and OS was significantly better for APP patients younger than 2 years at diagnosis (Fig. 3c, d). However, as mitoses are a primary distinguishing feature for this classification, and mitoses are more common in all tissues of infants, the finding might rather reflect the definition of the histological classification, rather than a deep biological principle in choroid plexus tumors [20, 31]. Deferring adjuvant treatment may be justified in select infants with APP and residual tumor [32].

In contrast to common belief [11, 22, 23], this prospective study did not confirm the impact of complete resection in CPC. This is likely the result of improved non-surgical treatment, and the data advocate for staged surgery in the context of comprehensive treatment concepts.

The use of chemotherapy in the treatment of choroid plexus tumors has increased since CPT-SIOP-2000 was designed [17, 21, 33–40] (Table 3). The treatment intensity of many of these protocols is higher than CPT-SIOP-2000, the patient numbers smaller, and the outcome similar [17, 35, 39]. There is no FDA-approved pharmacologic agent that is specific for CPT. A quantitative literature review comparing chemotherapeutic agents suggested benefit of etoposide, carboplatin, cyclophosphamide and vincristine, while similar suggestive evidence was absent for cisplatin, procarbazine and ifosfamide [25]. Since then, methotrexate has been added to the spectrum [19, 33, 34, 37]. CPT-SIOP-2000 adds evidence in support of the use of carboplatin (CarbEV) to achieve superior efficacy (significant for PFS, but not OS) compared to cyclophosphamide (CycEV), however the randomization numbers were low. Long recruitment time and small numbers of randomized patients are potential weakness in the study.

**Table 3:** Published studies on CPC and outcome

References	n CPC	Chemotherapy	Outcome 5y EFS/PFS	Outcome 5y OS	Comments
CPT-SIOP-2000 (this publication)	57	CarbEV/CycEV, and other (including registry patients)	41% EFS	65% OS (med f/u 6.0 y)	12 alive RT-free; 5 alive with RT at relapse; 6 LFS
Liu (2021) (SJYC07) [17]	13	HDMTX/VCR/Cis/Cy/(VBL)	61% PFS	68%	8 alive (3 with RT); 4 LFS
Siegfried (2017) [19]	22	CarbEV/ VEC/ ICE; BB-SFOP	25% EFS	64.7%	RT in 9; 5 alive RT-free, 1 XRT at relapse
Bahar (2017); Cleveland Clinic [30]	7	SIOP 2009 CarbEV/CycEV/IT/ HDMTX	3 relapse (all salvaged: CSRT and chemo)	100% (med f/u 5y)	1 adult (transformed CPP), med AAD 4.5 y, 2 M + , RT in 5 (3 at relapse)
Zaky (2015); HS I-III [31]	12	HS I-III	38% PFS	62% OS 5 alive RT-free, 1 RT at relapse	RT in 5 (4 at relapse, 1 focal RT at relapse)
Dudley (2015); SEER [6]	95			60% OS (med f/u 40 months)	RT in 16% GTR and RRT ns
Koh (2014); Seoul [32]	8	Carb/Cis/Cy/Ifo/VCR/VP16; 4 HDCT	2y PFS 0%	2 y OS 42%	RT in 4; 3 survived (med f/u 1.5 y) all HDCT, 2 foc RT
Bettegowda (2012); Johns Hopkins [33]	7	not detailed		71% (5 of 7 patients survived)	6 chemo, 3 RT
Grundy (2010); UKCCSG [34]	15	Carb/VCR/Cis/MTX	21.7% EFS	21.5% OS	ph II trial, 11/14 PD on chemo; no RT until PD; 4 alive RT-free
Lafay-Cousin (2010); Sickkids [15]	12	ICE	53.3% PFS	74.1% OS	all survivors had GTR/ NTR and RT-free; 1 GTR and HDCT at relapse) RT in 3 at relapse/residual
Fouladi (2009) [37]	5	Carbo/Cy/VP16	60% PFS	80% OS	ph II, 1 M + , all GTR, RT for M + or PD; 1 DOD, 1 died SNL
Geyer (2005); CCG 9921 [35]	9	VCR/Cis, Cy/VP16; Carb/Ifo/VP16; VCR/VP16, Carbo/VP16	33% 7 PD	63% (3 y) 4 patients died	ph II random no upfront RT
Chow (1999); SJCRH [36]	10	Cy/VP16/VCR/Cis, Carbo	3 PD	3 alive with RT 2 died	RT in 5 (3 at relapse)

*AAD* age at diagnosis, *Carb* carboplatin, *Cis* cisplatin, *csRT* craniospinal RT, *Cy* cyclophosphamide, *DOD* dead of disease, *GTR* gross total resection, *HDMTX* high-dose methotrexate, *HS* Head Start, *ICE* ifosfamide, carboplatin, etoposide, *Ifo* ifosfamide, *M* + metastatic, *med f/u* median follow-up, *NTR* near total resection; *PD* progressive disease, *RT* radiotherapy, *SNL* secondary neoplasm, *VBL* vinblastine; *VCR* vincristine; *VEC* vinctistine/etoposide/cyclophosphamide; *VP16* etoposide, *y* year

The efficacy of irradiation has been suggested in retrospective analyses [22, 23]. This study confirmed a trend toward longer survival. However, assignment of irradiation remains constrained due to the well-known late neuropsychological sequelae in younger children. Furthermore, particularly in the context of LFS, second malignancies remain a concern. A recent literature review was inconclusive with respect to specific indications for chemotherapy and radiotherapy [13].

Establishing a treatment algorithm as guidance was a major objective of the CPT-SIOP-2000 study, and the algorithm developed was widely followed in the international pediatric neuro-oncology community. Comparing the overall outcome of this study to the original literature analysis suggests a benefit of a structured algorithm in that the 2-year survival rate in the historical data collection was only half of what was found in CPT-SIOP-2000 [15, 19, 21, 23, 25]. Subsequent guidelines were more detailed and included response to treatment and LFS status [28, 30].

## Conclusion

CPT-SIOP-2000 demonstrates the feasibility of an international randomized clinical trial. CarbEV is effective and tolerable when nested in a multidisciplinary guideline framework. The robust findings of this study add long-term survival data as a benchmark for future intervention, and will help design risk-stratified guidelines.

## Supplementary Information

Below is the link to the electronic supplementary material.

Supplementary file1 (DOCX 512 kb)

## Data Availability

Not applicable.
