# Correction to: Final results of the Choroid Plexus Tumor Study CPT-SIOP-2000

**DOI:** 10.1007/s11060-025-05298-1

**Published:** 2025-10-20

**Authors:** Johannes E. Wolff, Stefaan W. Van Gool, Tezer Kutluk, Blanca Diez, Rejin Kebudi, Beate Timmermann, Miklos Garami, Jaroslav Sterba, Gregory N. Fuller, Brigitte Bison, Uwe R. Kordes

**Affiliations:** 1 University of Regensburg, Regensburg, Germany; 2 Present Address: Oncology Development, AbbVie, Chicago, IL USA; 3 IOZK Immune Oncological Centre Cologne, Cologne, Germany; 4 Department of Pediatric Oncology, Hacettepe University Medical School and Cancer Institute, Ankara, Turkey; 5 Neuro Oncology Program, Institute of Neurological Research, FLENI, Buenos Aires, Argentina; 6 Pediatric Hematology-Oncology, Oncology Institute, Istanbul University, Istanbul, Turkey; 7 Department of Particle Therapy, University Hospital Essen, West German Proton Therapy Centre Essen (WPE), West German Cancer Center (WTZ), German Cancer Consortium (DKTK), Essen, Germany; 8 2nd Department of Pediatrics, Semmelweis University, Budapest, Hungary; 9 Department of Pediatric Oncology, University Hospital Brno and Faculty of Medicine, Masaryk University, Brno, Czech Republic; 10 International Clinical Research Center, St. Anne’s University Hospital, Brno, Czech Republic; 11 Departments of Neuropathology and Neuroradiology, MD Anderson Cancer Center, Houston, USA; 12 Department of Neuroradiology, University Hospital of Augsburg, Augsburg, Germany; 13 Department of Pediatric Hematology and Oncology, University Medical Center Hamburg, Hamburg, Germany

**Correction to: Journal of Neuro-Oncology (2022) 156:599–613**

10.1007/s11060-021-03942-0

In this article, we have identified a minor numerical error in the second subsection in the **Methods and materials**; see the bold text in the incorrect and correct sentences specified below. It affects the accuracy of the article, but does not invalidate the conclusion. The corresponding supplemental figures were all correct.

**Incorrect**:

“Six cycles of chemotherapy were repeated every 28 days and consisted of etoposide 100 mg/msq on days 1–5, with vincristine 1.5 mg/msq on **day 1**. The third drug was randomized to either carboplatin 350 mg/msq on **days 1 and 2** (Supplemental Fig. 1a: CarbEV) or cyclophosphamide 1 g/msq on **days 1 and 2** (Supplemental Fig. 1b: CycEV).”

**Correct**:

“Six cycles of chemotherapy were repeated every 28 days and consisted of etoposide 100 mg/msq on days 1–5, with vincristine 1.5 mg/msq on **day 5**. The third drug was randomized to either carboplatin 350 mg/msq on **days 2 and 3** (Supplemental Fig. 1a: CarbEV) or cyclophosphamide 1 g/msq on **days 2 and 3** (Supplemental Fig. 1b: CycEV).”

The original article has been corrected.

