# Supplementary material for: Final results of the Choroid Plexus Tumor study CPT-SIOP-2000
Source: J Neurooncol. 2022 Jan 8;156(3):599–613. doi: 10.1007/s11060-021-03942-0 (PMC8860833; doi:10.1007/s11060-021-03942-0)
Supplement: Supplementary file 1 — Supplementary file1 (DOCX 512 kb) [file 11060_2021_3942_MOESM1_ESM.docx]

# Appendix: Supplemental Information

**Supplemental Table 1 a)** CPT-SIOP-2000 Radiotherapy Indication, Volume Prescriptions

**Supplemental Table 1 b)** CPT-SIOP-2000 Radiotherapy Dose-Volume Prescriptions

**Supplemental Table 2)** Toxicity Grading

**Supplemental Table 3)** Adverse Events Reported with 2 Cycles CPT-SIOP-2000 Chemotherapy

**Supplemental Table 4)** List of CPT-SIOP-2000 Study and Registry Sites, Investigators, number of recruited patients

**Supplemental Figure 1 a)** CPT-SIOP-2000 CarbEV Arm

**Supplemental Figure 1 b)** CPT-SIOP-2000 CycEV Arm

**Supplemental Figure 2)** Effect of Extent of Primary Resection in all 57 CPC

# Supplemental Table 1 a) Radiotherapy Indication, Volume Prescriptions

Age limitation: Radiotherapy only after 3^rd^ birthday

| **Histology (ICD-O code)** | **Metastases** | **Post-op Tumor** | **Response to chemotherapy (after 2 cycles)** | **Local Irradiation: Tumor (+Met)** | **Craniospinal Irradiation** |
| --- | --- | --- | --- | --- | --- |
| CPP (9390/0) | No | No |  | No | No |
|  | No | Yes | Regardless | No | No |
|  | Yes | Regardless | Regardless | Yes | No |
|  |  |  |  |  |  |
| APP (9390/1) | No | No |  | No | No |
|  | No | Yes | Regardless | Yes | No |
|  | Yes | Regardless | CR or PR | Yes | Yes |
|  | Yes | Regardless | SD or PD | Yes | Yes |
|  |  |  |  |  |  |
| CPC (9390/3) | No | No |  | Yes | No |
|  | No | Yes | CR or PR | Yes | No |
|  | No | Yes | SD or PD | Yes | Yes |
|  | Yes | Regardless | Regardless | Yes | Yes |

# Supplemental Table 1 b) Radiotherapy Dose-Volume Prescriptions

| **Craniospinal Irradiation** | | | | |
| --- | --- | --- | --- | --- |
| Part of CNS | Number of fractions | Dose per fraction | Total dose | Duration (weeks) |
| Cerebrum | 22 | 1.6 Gy | 35.2 Gy | 4.5 |
| Spinal axis | 22 | 1.6 Gy | 35.2 Gy | 4.5 |
| Tumor/ boost | + 11 | 1.8 Gy | + 19.8 Gy | + 2.2 |
| Metastases | + 8 | 1.8 Gy | + 14.4 Gy |  |
| Total/ tumor | 25 |  | 55 Gy |  |
| Total/ metastases |  |  | 49.6 Gy |  |

| **Irradiation of Tumor Sites** | | | | |
| --- | --- | --- | --- | --- |
| Part of CNS | Number of fractions | Dose per fraction | Total dose | Duration (weeks) |
| Tumor region | 30 | 1.8 Gy | 54.0 Gy | 6 |

# Supplemental Table 2) Toxicity Grading


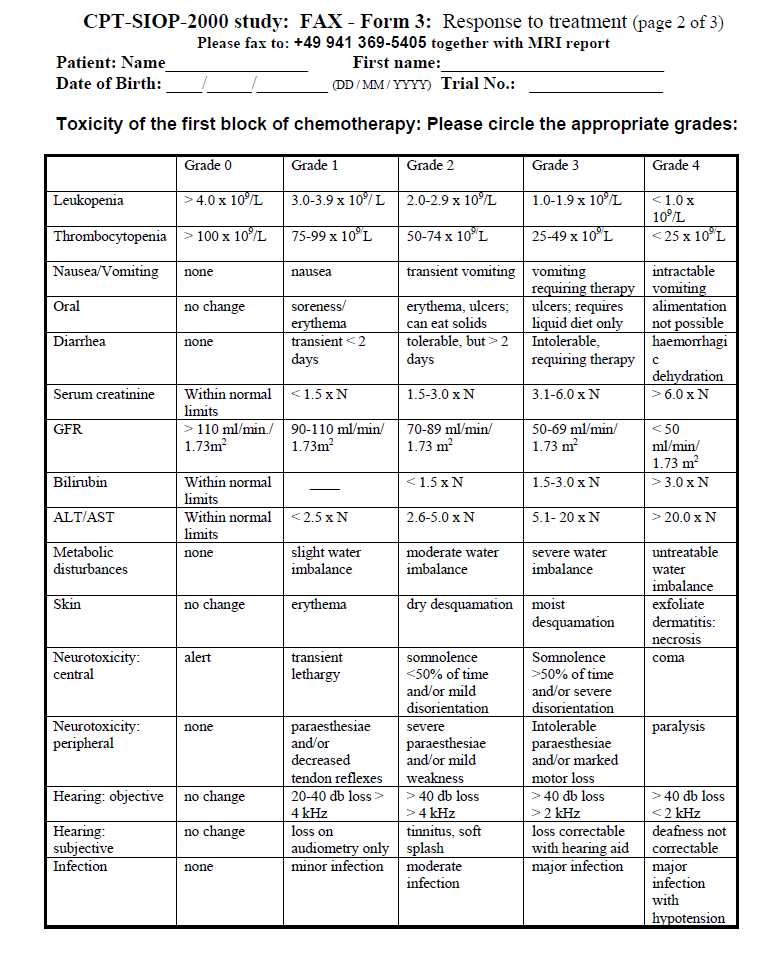


# Supplemental Table 3) Adverse Events Reported with 2 Cycles of Chemotherapy

|  | **CarbEV arm, n=31 patients** | | | | | **CycEV arm, n=31 patients** | | | | |
| --- | --- | --- | --- | --- | --- | --- | --- | --- | --- | --- |
| Grade | 0 | 1 | 2 | 3 | **4** | **0** | **1** | **2** | **3** | **4** |
| Leukopenia | 2 | 0 | 1 | 4 | 24 | 4 | 2 | 7 | 15 | 3 |
| Thrombocytopenia | 11 | 4 | 5 | 3 | 7 | 7 | 4 | 0 | 6 | 14 |
| Nausea/Vomiting | 7 | 7 | 13 | 4 | 0 | 13 | 5 | 9 | 4 | 0 |
| Oral mucositis | 23 | 3 | 5 | 0 | 0 | 22 | 7 | 0 | 1 | 1 |
| Diarrhea | 19 | 5 | 7 | 1 | 0 | 19 | 11 | 1 | 0 | 0 |
| Creatinine | 31 | 0 | 0 | 0 | 0 | 28 | 2 | 0 | 0 | 0 |
| GFR | 10 | 3 | 1 | 0 | 0 | 15 | 4 | 1 | 0 | 0 |
| Bilirubin | 29 | 0 | 1 | 0 | 0 | 26 | 0 | 1 | 0 | 0 |
| ALT/AST | 22 | 4 | 4 | 0 | 0 | 12 | 11 | 4 | 2 | 0 |
| Metabolic Disturbances | 29 | 2 | 0 | 0 | 0 | 29 | 1 | 1 | 0 | 0 |
| Skin | 22 | 8 | 2 | 0 | 0 | 29 | 2 | 0 | 0 | 0 |
| CNS | 28 | 2 | 0 | 0 | 0 | 28 | 3 | 0 | 0 | 0 |
| PNS | 29 | 1 | 0 | 0 | 0 | 28 | 2 | 0 | 0 | 0 |
| Hearing: objective | 13 | 1 | 0 | 0 | 0 | 17 | 0 | 0 | 0 | 0 |
| Hearing: subjective | 26 | 0 | 2 | 0 | 0 | 24 | 0 | 0 | 0 | 0 |
| Infection | 12 | 5 | 9 | 5 | 0 | 14 | 10 | 6 | 1 | 0 |

Data collected after cycle 1 and cycle 2 of each chemotherapy arm, both from study and non-study patients. Bone marrow toxicity and infections were more common with cyclophosphamide. Nausea, vomiting and skin toxicity were more common with carboplatin. There was no treatment related death. CNS: Central Nervous System toxicity; GFR: Glomerular Filtration Rate; PNS: Peripheral Nervous System toxicity

# Supplemental Table 4) List of Sites, Investigators, Number of Recruited Patients

Definition of study site and investigator for CPT-SIOP-2000 pre-dates the Clinical Trials Directive 2001/20/EC that went into effect 2004.

| **Country code** | **Site** | **Investigator** | **patients** |
| --- | --- | --- | --- |
| AR | Buenos Aires | Diez | 2 |
| AR | Buenos Aires | Negro | 1 |
| AT | Graz | Benesch | 1 |
| AT | Linz | Ebetsberger | 5 |
| AT | Wien | Slavc | 3 |
| AT | Salzburg | Sperl | 1 |
| AU | Auckland | Law (surgeon) | 1 |
| AU | Brisbane | Hassall | 2 |
| AU | Brisbane | Irving | 1 |
| AU | Melbourne | Cantsilieris (RN) | 1 |
| AU | Melbourne | Hassall | 1 |
| AU | Melbourne | Maixner (surgeon) | 1 |
| AU | Montreal/ Mc Gill | Renaud (RN) | 1 |
| AU | Perth | Rowlands (RN) | 1 |
| AU | Subiaco/ Perth | Kendrew (RN) | 1 |
| BE | Ghent | Vandecruys | 2 |
| BE | Leuven | Van Gool | 3 |
| BY | Minsk | Konoplya | 3 |
| CA | Montreal | Moghrabi | 6 |
| CH | Basel | Kühne | 1 |
| CH | St. Gallen | Hengartner | 1 |
| CH | Zurich | Gerber | 1 |
| CL | Santiago | Quintana | 2 |
| CZ | Brno | Pavelka | 5 |
| CZ | Prag | Sumerauer | 4 |
| DE | Aachen | Mertens | 2 |
| DE | Augsburg | Gnekow | 1 |
| DE | Berlin (Buch) | Kiwit | 1 |
| DE | Berlin (Charité Virchow) | Hernaíz-Driever | 1 |
| DE | Bielefeld | Jorch | 1 |
| DE | Bielefeld | Otte | 2 |
| DE | Bonn | Calaminus | 1 |
| DE | Braunschweig | Eberl | 2 |
| DE | Bremen | Pekrun | 2 |
| DE | Cologne | Berthold | 1 |
| DE | Cologne | Simon | 1 |
| DE | Dortmund | Schneider | 1 |
| DE | Dresden | Gahr | 1 |
| DE | Dresden | Smitka | 1 |
| DE | Dresden | Suttorp | 2 |
| DE | Düsseldorf | Göbel | 2 |
| DE | Frankfurt | Klingebiel | 1 |
| DE | Freiburg | Kontny | 1 |
| DE | Freiburg | Omran | 1 |
| DE | Gießen | Reiter | 1 |
| DE | Göttingen | Lakomek | 3 |
| DE | Greifswald | Lode | 1 |
| DE | Halle | Körholz | 2 |
| DE | Halle | Kramm | 1 |
| DE | Hamburg | Kordes | 1 |
| DE | Heidelberg | Witt | 5 |
| DE | Homburg | Graf | 1 |
| DE | Karlsruhe | Leipold | 2 |
| DE | Kiel | Schrappe | 1 |
| DE | Kiel | Suttorp | 1 |
| DE | Ludwigsburg | Walka | 1 |
| DE | Magdeburg | Vorwerk | 1 |
| DE | Mainz | Dittrich | 1 |
| DE | Mainz | Gutjahr | 3 |
| DE | Munich (Hauner) | Peraud (surgeon) | 1 |
| DE | Munich (Hauner) | Schmid | 3 |
| DE | Munich (LMU) | Haas | 1 |
| DE | Munich (TUM) | Burdach | 1 |
| DE | Munich (TUM) | Nathrath | 2 |
| DE | Münster | Sträter | 1 |
| DE | Regensburg | Peters | 3 |
| DE | Regensburg | Wolff | 1 |
| DE | St. Augustin | Reinhard | 2 |
| DE | Stuttgart | Maass | 2 |
| DE | Trier | Feddersen | 1 |
| DE | Tübingen | Handgretinger | 1 |
| DE | Würzburg | Schlegel | 3 |
| DE | Würzburg | Schropp | 2 |
| ES | Barcelona | Cruz | 2 |
| ES | Malaga | Herrero | 1 |
| ES | Valencia | Canete | 2 |
| HU | Budapest | Hauser | 1 |
| IL | Petach Tikuva | Toledano | 1 |
| IT | Genoa | Garre | 1 |
| IT | Milan | Massimino | 2 |
| MX | Mexico City | Medina-Sanson | 1 |
| MY | Kuala Lumpur | Ariffin | 1 |
| NL | Rotterdam | Blink | 1 |
| NL | Rotterdam | Pieters | 1 |
| NL | Rotterdam | Reddinguis | 1 |
| NZ | Christchurch | Corbett | 1 |
| PT | Lisboa | Nunes | 1 |
| SE | Lund | Colberger-Rudbäck | 1 |
| SI | Ljubljana | Kitanovski | 1 |
| SK | Bratislava | Puskacova | 2 |
| TR | Ankara | Kutluk | 5 |
| TR | Istanbul | Kebudi | 1 |
| TR | Istanbul (Marmara) | Berrak | 1 |
| US | Houston | Hetherington | 1 |
| US | Houston | Wolff | 3 |
| US | New York | Finlay | 1 |
|  | **Total unique: 80** | **Total unique: 93** | **158** |

# Supplemental Figure 1 a) CarbEV Arm


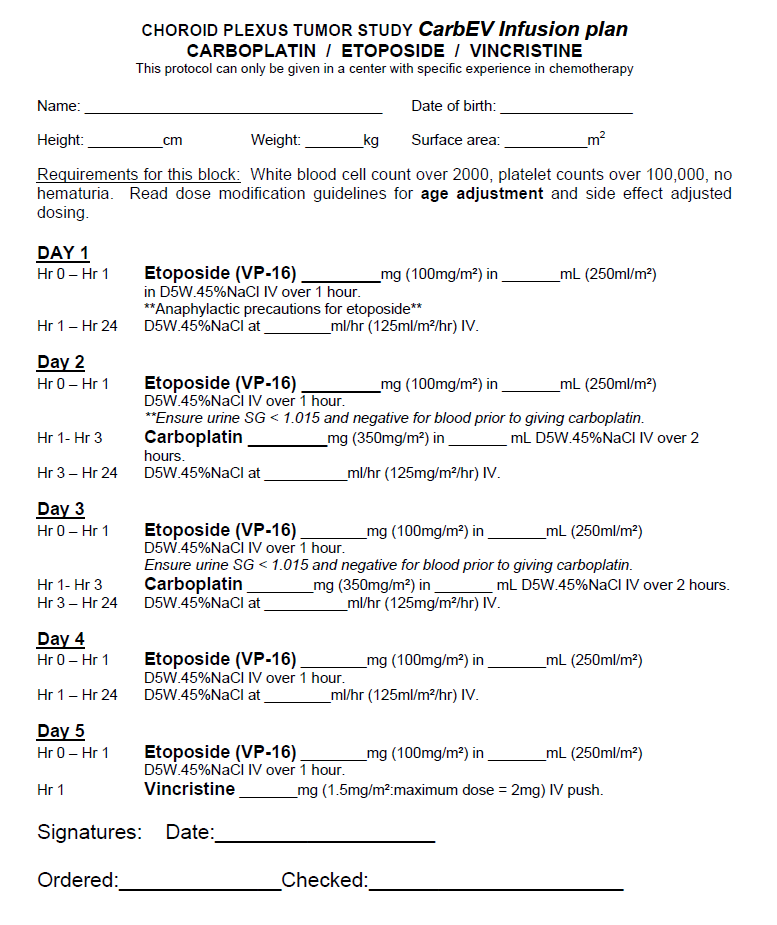


# Supplemental Figure 1 b) CycEV Arm


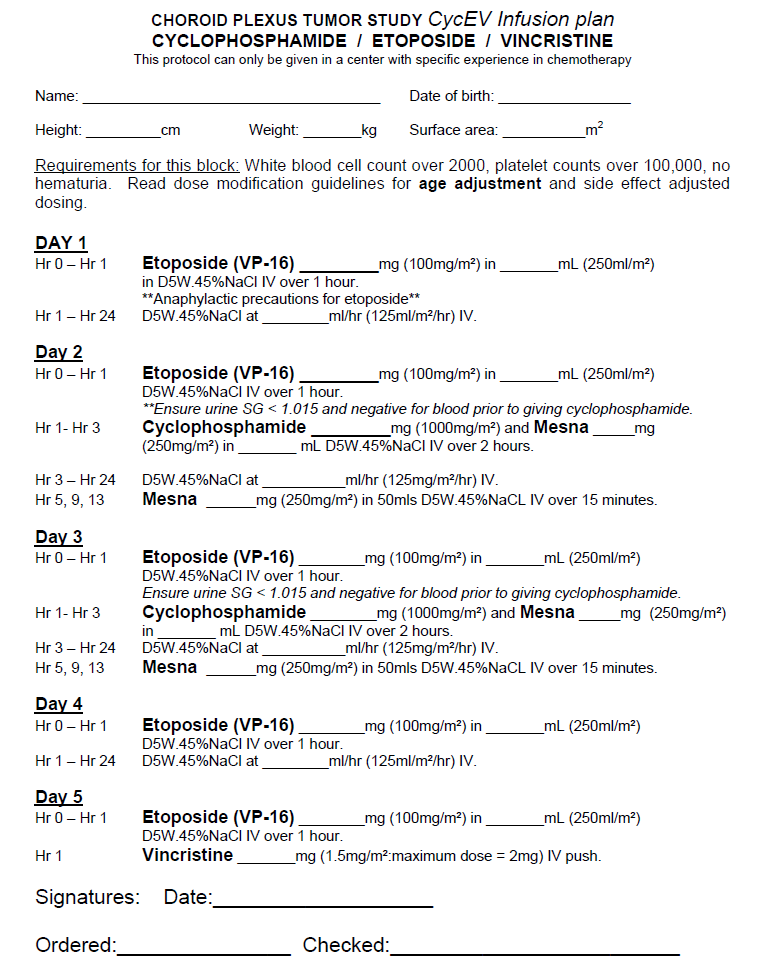


# Supplemental Figure 2) Effect of Extent of Primary Resection in all 57 CPC


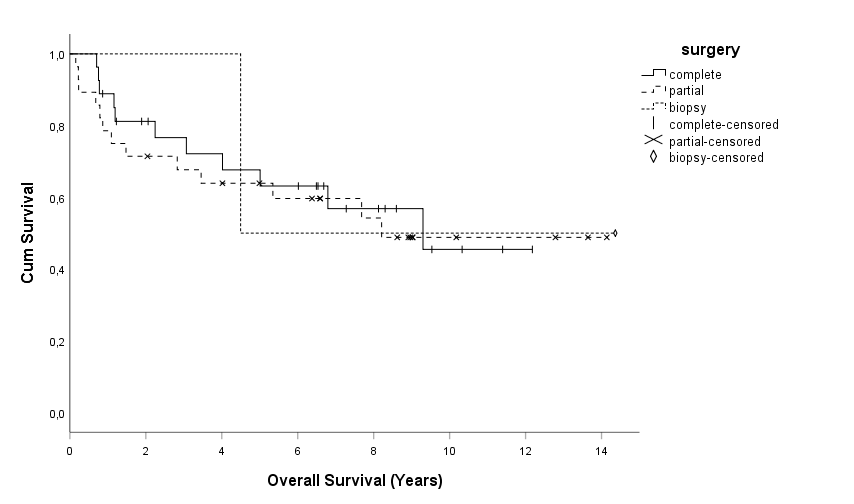


**Number at risk**

complete 17 19 16 14 8 3 1 0

partial 28 20 17 14 10 5 3 1

biopsy 2 2 2 1 1 1 1 1

Including metastatic and non-metastic CPC
